# Brain Circuit Alterations and Cognitive Disability in Late-Onset Cobalamin D Disorder

**DOI:** 10.3390/jcm9040990

**Published:** 2020-04-02

**Authors:** Javier De Las Heras, Ibai Diez, Antonio Jimenez-Marin, Alberto Cabrera, Daniela Ramos-Usuga, Marta Venecia Diaz-Fernandez, Leire Torices, Caroline E. Nunes-Xavier, Rafael Pulido, Juan Carlos Arango-Lasprilla, Jesus M. Cortes

**Affiliations:** 1 Division of Pediatric Metabolism, Cruces University Hospital, 48903 Barakaldo, Spain; javier.delasherasmontero@osakidetza.eus (J.D.L.H.); martavenecia.diazfernandez@osakidetza.eus (M.V.D.-F.); 2 Inborn Errors of Metabolism Group, Biocruces Bizkaia Health Research Institute, 48903 Barakaldo, Spain; 3 Department of Pediatrics, University of the Basque Country (UPV/EHU), 48940 Leioa, Spain; 4 Gordon Center for Medical Imaging, Department of Radiology, Massachusetts General Hospital and Harvard Medical School, Boston, MA 02115, USA; idiezpalacio@mgh.harvard.edu; 5 Athinoula A. Martinos Center for Biomedical Imaging, Massachusetts General Hospital, Harvard Medical School, Boston, MA 02129, USA; 6 Neurotechnology Laboratory, Tecnalia Health Department, 48160 Derio, Spain; 7 Computational Neuroimaging Group, Biocruces Bizkaia Health Research Institute, 48903 Barakaldo, Spain; antonio.jimenezmarin@osakidetza.eus; 8 Biomedical Research Doctorate Program, University of the Basque Country (UPV/EHU), 48940 Leioa, Spain; daniela.ramosusuga@osakidetza.eus; 9 Osatek, 48011 Vitoria-Gazteiz, Spain; alberto.cabrerazubizarreta@osakidetza.eus; 10 Biocruces Bizkaia Health Research Institute, 48903 Barakaldo, Spain; 11 Biomarkers in Cancer Unit, Biocruces Bizkaia Health Research Institute, 48903 Barakaldo, Spain; leire.toricescabarcos@osakidetza.eus (L.T.); caroliten@gmail.com (C.E.N.-X.); rpulidomurillo@gmail.com (R.P.); 12 Department of Tumor Biology, Institute for Cancer Research, Oslo University Hospital Radiumhospitalet, 0424 Oslo, Norway; 13 IKERBASQUE. Basque Foundation for Science, 48013 Bilbao, Spain; 14 Department of Cell Biology and Histology, University of the Basque Country (UPV/EHU), 48940 Leioa, Spain

**Keywords:** cobalamin, vitamin B12, cobalamin D disease, methylmalonic aciduria and homocystinuria, resting state, functional neuroimaging, diffusion imaging, cognitive functioning

## Abstract

Neuroimaging studies describing brain circuits’ alterations in cobalamin (vitamin B12)-deficient patients are limited and have not been carried out in patients with inborn errors of cobalamin metabolism. The objective of this study was to assess brain functionality and brain circuit alterations in a patient with an ultra-rare inborn error of cobalamin metabolism, methylmalonic aciduria, and homocystinuria due to cobalamin D disease, as compared with his twin sister as a healthy control (HC). We acquired magnetic resonance imaging (including structural, functional, and diffusion images) to calculate brain circuit abnormalities and combined these results with the scores after a comprehensive neuropsychological evaluation. As compared with HC, the patient had severe patterns of damage, such as a 254% increment of ventricular volume, pronounced subcortical and cortical atrophies (mainly at striatum, cingulate cortex, and precuneus), and connectivity alterations at fronto-striato-thalamic circuit, cerebellum, and corpus callosum. In agreement with brain circuit alterations, cognitive deficits existed in attention, executive function, inhibitory control, and mental flexibility. This is the first study that provides the clinical, genetic, neuroanatomical, neuropsychological, and psychosocial characterization of a patient with the cobalamin D disorder, showing functional alterations in central nervous system motor tracts, thalamus, cerebellum, and basal ganglia, that, as far as we know, have not been reported yet in vitamin B12-related disorders.

## 1. Introduction

Cobalamin (cbl), or vitamin B12, is a water-soluble vitamin present in animal products such as meat, fish, eggs, and milk [1]. Nutritional and acquired cbl deficiencies, as well as inborn errors of cbl absorption and intracellular trafficking, cause multisystem disease with important neurological involvement [2,3]. 

Inborn errors of cbl—cofactor synthesis comprise a group of rare disorders affecting multiple steps between the lysosomal release of cbl and the synthesis of adenosylcobalamin in the mitochondria and methylcobalamin in the cytosol [4]. To date, nine distinct defects have been identified: cblA–cblG, cblJ, and mut [5,6]. The cobalamin D (cblD) disorder is an autosomal recessive disease of cbl metabolism caused by mutations in the *MMADHC* gene [4] that can result in isolated homocystinuria, isolated methylmalonic aciduria, or the combined form, methylmalonic aciduria and homocystinuria (MMA/HC) [7]. Only a few patients with cblD defect have been described because it is one of the rarest inborn disorders of cbl metabolism. Most patients affected by inborn errors of cbl metabolism present in the neonatal period with seizures, hypotonia, lethargy megaloblastic anemia, and developmental delay [8]. Late-onset forms are extremely rare and difficult to diagnose because they may present with rather nonspecific psychiatric and behavioral symptoms along with different degrees of cognitive impairment [9].

Brain abnormalities are common in inborn errors of cbl metabolism and include white matter changes, basal ganglia lesions, hydrocephalus, and diffuse cerebral atrophy with enlargement of extra-axial spaces [10]. Along with morphological alterations, cognitive dysfunction, behavioral and psychiatric problems are frequent in late-onset forms [2].

While several studies have investigated the neurological implications of vitamin B12 deficiency focusing on the role of biomarkers like homocysteine and methylmalonic acid [10,11,12], neuroimaging studies describing brain circuits´ alterations in vitamin B12-deficient patients are limited [13]. To the best of our knowledge, such studies have not been carried out in patients with inborn errors of cbl metabolism.

Thus, the purpose of the present study is to describe the clinical, imagining, genetic, cognitive, and emotional characteristics of a patient recently diagnosed with late-onset combined Cobalamin D disease and compare them to the characteristics of a healthy control, his twin sister.

## 2. Experimental Section

### 2.1. In Vitro Analysis of the MMADHC Variants

A pCMV3 *MMADHC*-GFP Spark plasmid containing the human *MMADHC* cDNA (nucleotide entry NM_015702; protein entry NP_056517) was purchased from Sino Biologicals, and the pRK5 HA-*MMADHC* plasmid was made by subcloning of the *MMADHC* cDNA (from pCMV3 *MMADHC*-GFP Spark) into the pRK5 HA plasmid [14], removing the C-terminal GFP and adding an N-terminal hemagglutinin (HA) epitope. The F161fsX14 and R250X *MMADHC* variants were made on pRK5 HA-*MMADHC* by site-directed mutagenesis, as described [15], and mutations were verified by DNA sequencing. For ectopic expression of the *MMADHC* variants, simian kidney COS-7 cells were transfected with pRK5 HA-*MMADHC* plasmids using GenJet transfection reagent, following the manufacturer’s indications. Immunoblotting was performed as described [16]. Briefly, 48 h after transfection, whole-cell protein extracts were prepared by cell lysis in ice-cold M-PER^TM^ lysis buffer (Thermo Fisher Scientific, Walthham, MA, USA) supplemented with PhosSTOP phosphatase inhibitor and complete protease inhibitor cocktails (Roche, Basel, Switzerland), followed by centrifugation at 15,200× *g* for 10 min and collection of the supernatant. Then, 50 μg of proteins were resolved in 10% SDS-PAGE under reducing conditions and transferred to PVDF membranes (Immobilon-FL, Millipore, MA, USA). Immunoblotting was performed using an anti-HA 12CA5 monoclonal antibody (Sigma-Aldrich, Saint-Louis, MI, USA), followed by anti-mouse IgG-IRDyeR 800CW (LI-COR Biosciences, Lincoln, NE, USA) secondary antibodies. 

### 2.2. Magnetic Resonance Imaging

#### 2.2.1. Imaging Acquisition

Brain MRI was acquired in a Philips 3-Tesla Achieva Dstream MRI scanner with a 32-channel head coil. High-resolution structural MRI was acquired using a T1-weighted 3D turbo field echo (TFE) with the following parameters: repetition time (TR) = 7.4 ms, echo time (TE) = 3.4 ms, voxel size = 1.1 × 1.1 × 1.2 mm^3^, slice thickness = 1.2 mm, field of view (FOV) =  250 × 250 mm^2^, 300 contiguous sagittal slices covering the entire brain and brain stem. Diffusion-weighted images were acquired using pulsed spin-echo single-shot echo-planar imaging (SE-EPI) under the following parameters: TR  =  7540 ms, TE  =  77 ms, voxel size= 2 × 2 × 2 mm^3^, slice thickness = 2  mm, no gap between slices, FOV = 240  ×  240 mm^2^, SENSitivity Encoding (SENSE) acceleration factor = 3, 65 contiguous sagittal slices covering the entire brain and brainstem. A diffusion gradient was applied along 32 noncollinear directions with a b value of 1000 s/mm^2^. Additionally, one set of images was acquired without diffusion weighting (b = 0 s/mm^2^). Functional images assessing changes in blood-oxygenation-level-dependent (BOLD) T2* signals were obtained with an interleaved echo-Planar Imaging EPI sequence using SENSE (with a factor of 2.2). The subjects lay quietly for 7.40 minutes, during which 214 whole-brain volumes were obtained under the following parameters: TR  =  2100 ms, TE  =  27 ms; FOV = 240  ×  240 mm^2^; voxel size = 3 × 3 × 3 mm^3^, slice thickness = 3  mm, 80  ×  80 pixel matrix, 45 axial slices, interleaved in ascending order.

#### 2.2.2. Imaging Analyses

Structural alterations were assessed by voxel-based morphometry (VBM) applied with FMRIB Software Library (FSL)-VBM [17], an optimized VBM protocol carried out with FSL tools. First, by using the structural images, the skull was removed and gray matter was segmented before being registered to the MNI152 standard space with nonlinear registration. The resulting images were averaged and flipped along the x-axis to create a left–right symmetric, study-specific gray matter template. Second, all native gray matter images were nonlinearly registered to this study-specific template and “modulated” to correct for local expansion (or contraction) due to the nonlinear component of the spatial transformation. The modulated gray matter images were then smoothed with an isotropic Gaussian kernel of full width at half maximum of 9.42 mm. For calculation of ventricular volumes, Freesurfer (Version 5.3.0, Boston, MA, USA, https://surfer.nmr.mgh.harvard.edu/) was used. 

Circuits’ alterations corresponding to abnormalities from diffusion images were assessed using FSL (FMRIB Software Library, version 5.0, Oxford, UK) following a postprocessing procedure similar to that used in previous work [18,19,20]. First, an eddy current correction was applied to overcome the artifacts produced by variation in the direction of the gradient fields of the MR scanner, together with the artifacts produced by head movements. Next, the subject motion variable was extracted from the transformation applied by the eddy current correction from every volume to the reference volume (the first one, the b = 0 volume). Next, using the corrected data, a local fitting of the diffusion tensor was applied to compute the diffusion tensor model for each voxel. Using the diffusion tensor, we computed mean diffusivity maps only for regions belonging to white matter atlases [21,22]. These maps were transformed into Montreal Neurological Institute (MNI) space and smoothed with an isotropic Gaussian kernel with a sigma of 3 mm.

Functional alterations were assessed using FSL and AFNI (16.2.07, Milwaukee, WI, USA, http://afni.nimh.nih.gov/afni/) following a procedure similar to that used in previous work [19,20]. First, slice-time correction was applied to the functional MRI (fMRI). Next, each volume was aligned to the middle volume to correct for head movement artifacts. After intensity normalization, we removed the effect of confounding factors: movement time courses, the average cerebrospinal fluid signal, and the average white matter signal, followed by a band-pass filter between 0.01 and 0.08 Hz. The functional data were normalized to the MNI152 brain template, with a voxel size of 3 × 3 × 3 mm^3^ and spatially smoothed with a 6 mm full width at half maximum isotropic Gaussian kernel. In addition to head motion correction, we performed scrubbing, by which means all time points with a frame-wise displacement greater than 0.5 were interpolated by a cubic spline [23]. Variations with respect to the healthy control were calculated using the formula abs (XC-XP)/XC, where abs, C, and P indicate respectively absolute value, control, and patient. The same formula was applied for the three classes of analyses, simply considering X equal to volume for the structural images, equal to MD for the diffusion images, and equal to signal variance per voxel for the functional images. Tolerance thresholds were equal to 0.35 for the structural and diffusion images and equal to 0.20 for the functional images.

MRI abnormalities were matched to cognitive function by means of neurosynth maps, available at http://neurosynth.org and obtained after meta-analysis of more than 14,000 different task fMRI published studies and resulting in more than 150,000 different co-activation maps. In particular, we made use of the following “Term-based meta-analyses” search words: Recognition, Visuospatial, Memory, Interference, Attention, Learning, Recall, Stroop, Inhibition, Executive, Response Time, and Comprehension.

### 2.3. Neuropsychological Evaluation 

Both patient and healthy control underwent a comprehensive evaluation that included measures of visuospatial and visuoconstructional skills (Rey–Osterrieth Complex Figure Test [24]), attention (The Stroop Color and Word Test [25] and D2 Test of Attention [26]), executive functioning (Modified Wisconsin Card Sorting Test [27,28] (M-WCST) and Trail Making Test [29] (TMT)), speed of information processing (Symbol Digit Modalities Test [30] (SDMT)), language (Shortened Version of the Token Test [31], Controlled Oral Word Association Test [32] (COWAT), and Peabody Picture Vocabulary Test [33] (PPVT-III)), and memory (Learning and Verbal Memory Test [34] (TAMV-I)).

For psychosocial assessment the following tests were used: Children Depression Inventory [35] (CDI), Revised Children’s Manifest Anxiety Scale [36] (CMAS-R), Pediatric Quality of Life Inventory [37] (PedsQL) Parent-Report, and Adaptive Behavior Assessment System, Second Edition [38] (ABAS-II). All of these tests had normative data available for Spanish-speaking children and adolescents [39,40,41,42,43,44,45,46,47,48]. 

Patient vs. control neuropsychological discrepancies were assessed by calculating the differences in Z-score, specifically using the formula diff = abs (abs(ZC)-abs(ZP)) if both ZC and ZP had an equal sign (abs representing absolute values) or as diff = abs(abs(ZC) + abs(ZP)) for the case of both ZC and ZP having a different sign. 

### 2.4. Ethical Considerations

Informed consent was signed by the participants´ parents, and the children agreed after the objectives and procedures of the study were explained and their doubts resolved. The study was conducted in accordance with the Declaration of Helsinki, and the protocol was approved by the Ethical Committee at Cruces University Hospital (CEIC E17/26).

## 3. Results

### 3.1. Clinical Characterization

We present a case of a male, 11 years and 9 months old, who at 10 years and 5 months developed progressive encephalopathy with regression, i.e., deterioration in school performance, behavioral and personality changes, episodes of acute mental confusion and lethargy, and weight loss. Six months after the onset of the clinical manifestations, a left frontoparietal subdural hematoma was diagnosed. Neuropsychiatric symptoms persisted after surgical evacuation of the subdural hematoma [49]. At this point, enlarged subarachnoid spaces and cerebral ventricular system were evident by MRI [49]. Eleven months after the clinical onset, the patient was referred to our clinic. On our first evaluation, he presented an evident disorientation, inattention, and serious mental slowness. Laboratory tests were performed using standard hospital´s laboratory methods, including liquid chromatography–tandem mass spectrometry, and showed increased plasma homocysteine (144 µmol/L; normal value 5 to 15 µmol/L) and methylmalonic acid (67.8 µmol/L; normal value 0.08–0.58 µmol/L) levels, while methionine levels were within the normal range (18 µmol/L; normal value 7–47 µmol/L). Blood cell counts were within the normal range. Treatment with intramuscular OH-cobalamin (2 mg/day), oral betaine (250 mg/kg/day), and folinic acid (15 mg/day) was immediately initiated, with a rapid decrease in homocysteine and methylmalonic acid levels, and an increase in methionine levels (Table 1). MMA/HC due to cblD disease was confirmed by genetic testing, which showed a novel *MMADHC* mutation (c.438-442delAGAGT; p.F161fsX14) in heterozygosis with the mutation (c.748C > T, p.R250X). The patient´s twin sister, without any disease symptoms, underwent the same genetic testing, and no mutations were identified.

### 3.2. Genotype Characterization

**Figure 1:**
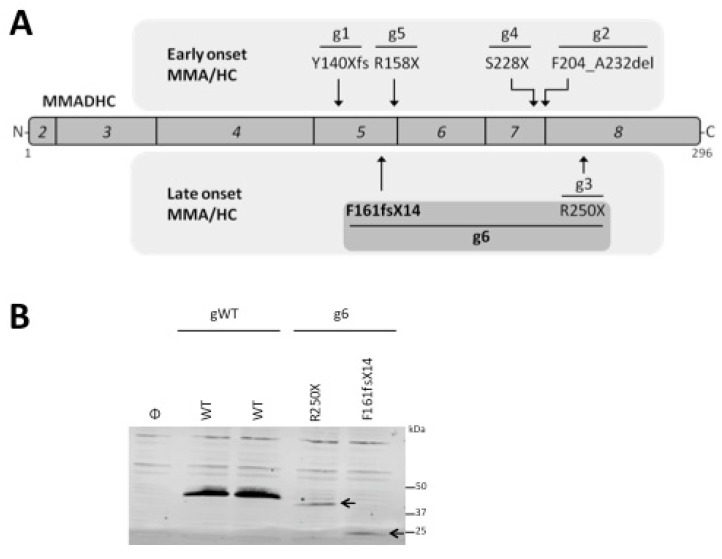
Genotype and protein dysfunction in combined Cobalamin D disorder: (**a**) Schematic depiction of the distinct *MMADHC* protein alterations and associated genotypes in patients with combined cblD defect. Exons (2 to 8, in italics) encoding *MMADHC* protein (amino acid 1 to 296, from N-terminus (N) to C-terminus(C)) are indicated. Protein alterations that result from *MMADHC* mutations causing early onset methylmalonic aciduria and homocystinuria (MMA/HC) (top) or late-onset MMA/HC (bottom) are shown, with an indication of the associated genotype (g1 to g6; see Table 2 for details). Note that g1 to g5 are homozygous genotypes for each mutation, whereas g6 (this study) is a heterozygous compound genotype that includes the novel allelic variant encoding F161fsX14 in combination with the allelic variant encoding R250X. Amino acids are indicated by the single-letter code; X, stop codon; fs, frameshift; del, deletion; (**b**) *MMADHC* recombinant protein variants from genotype WT (gWT) and genotype F161fsX14/R250X (g6). COS-7 cells were transfected with empty vector (Φ, control), or with plasmids containing the indicated HA-*MMADHC* variants, and cell lysates were resolved by SDS-PAGE under reducing conditions, followed by immunoblot using anti-HA (hemagglutinin) antibody. Arrows indicate the bands corresponding to the *MMADHC* protein variants R250X and F161fsX14.

The genotype reported in this study ((c.438-442delAGAGT; p.F161fsX14) in heterozygosis with (c.748C > T, p.R250X)) has not been described to the best of our knowledge. We have called this novel cblD MMA/HC as genotype g6, as compared with the previously described cblD MMA/HC genotypes (g1 to g5), summarized in Table 2. Unlike the other described homozygous genotypes, the g6 genotype is a heterozygous compound genotype which contains a novel allelic variant (c.438-442delAGAGT) encoding a F161fsX14 *MMADHC* protein variant, in combination with the allelic variant c.748C > T encoding the R250X *MMADHC* protein variant (Figure 1A). Immunoblot analysis using recombinant *MMADHC* proteins revealed that these mutations generate truncated *MMADHC* proteins, which were expressed at lower levels when compared with wild-type *MMADHC* proteins (Figure 1B). 

### 3.3. Imaging Characterization

**Figure 2:**
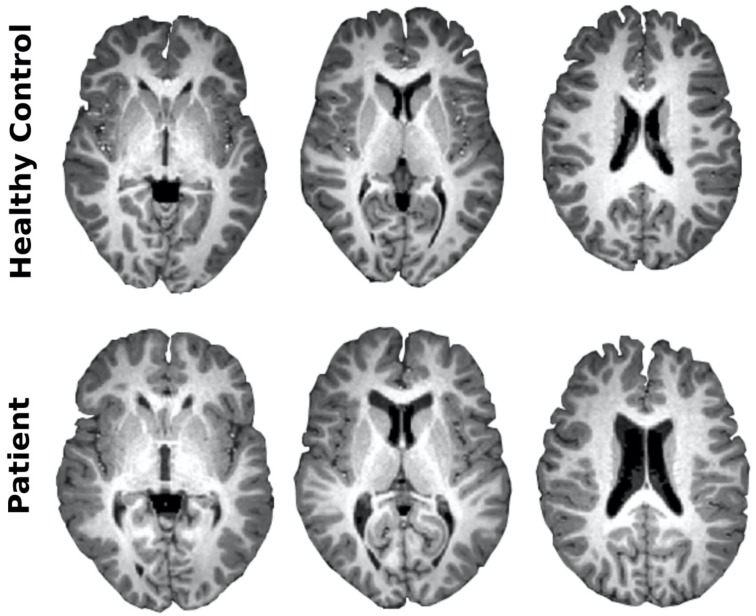
Large increased ventricular volume in combined Cobalamin D disorder. After acquiring structural brain images from both the healthy control and the patient with combined cblD disorder, the ventricular volume increased a proportion of 254%. Here, the same axial slices are shown for both healthy control and patient.

**Figure 3:**
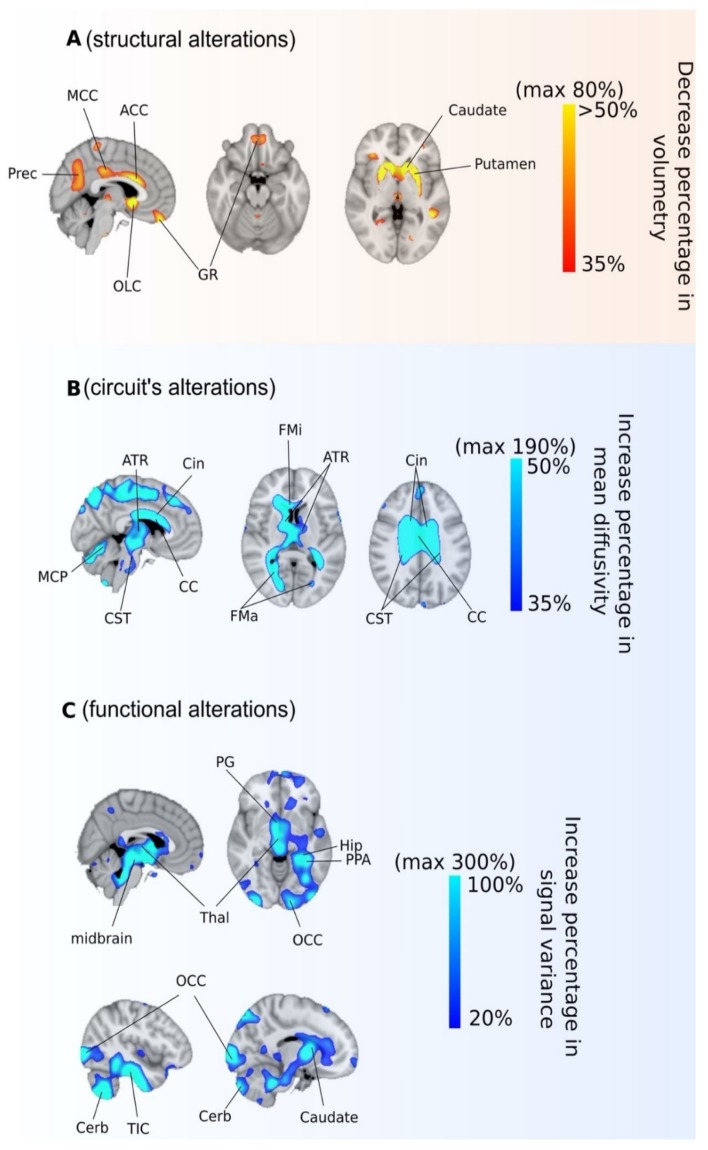
Morphological, circuits’, and functional alterations in combined Cobalamin D disorder. Three different MRI acquisitions and analyses provided three different classes of alterations: (**A**) structural; (**B**) circuits’; (**C**) functional. Abbreviated labels are OCC: Occipital cortex; Cerb: Cerebellum; TIC: Temporal inferior cortex; Hip: Hippocampus; Thal: Thalamus; PPA: Parahippocampal; Prec: Precuenus; GR: Gyrus rectus; CC: Corpus callosum; MCC: Midcingulate cortex; ACC: Anterior cingulate cortex; OLC: olfactory cortex; PG: pale globe; MCP: middle cerebellar peduncle; CST: cortico-spinal tract; ATR: anterior thalamic radiation; Cin: cingulum; FMa: forceps major; FMi: forceps minor. Orange and blue colors indicate respectively patient < control and patient > control.

The patient displayed severe morphological brain alterations. Perhaps the most remarkable change was an increase of ventricular volume of 254%, as compared with the healthy control (Figure 2). Quantitative analysis, e.g., voxel-based morphometry, provided ratios of volume decrements in several regions (Figure 3A), putamen (89%), globus pallidus (80%), caudate (75%), anterior (56%) and middle (43%) cingulate cortex, olfactory cortex (49%), precuneus (47%), and gyrus rectus (43%). No volume increments were found. 

After analyzing diffusion images (Figure 3B), mean diffusivity increases were found (revealing anatomical disconnectivity) from corpus callosum (193%), anterior thalamic radiation (140%), corticospinal tract (125%), forceps minor (110%), cingulum (105%), forceps major (100%), acoustic radiation (99%), inferior fronto-occipital fasciculus (98%), and medial lemniscus (92%). No reductions in mean diffusivity decrements corresponding to patient hyperconnectivity were found.

After analyzing functional images (Figure 3C), a variance increment of the blood-oxygen-level-dependent signal (indicating functional abnormalities) was found in brain stem (390%), caudate (340%), cerebellum (250%), fusiform (248%), parahippocampus (230%), inferior temporal cortex (225%), hippocampus (195%), occipital cortex (140%), and thalamus (125%). A variance decrease was not found.

**Figure 4:**
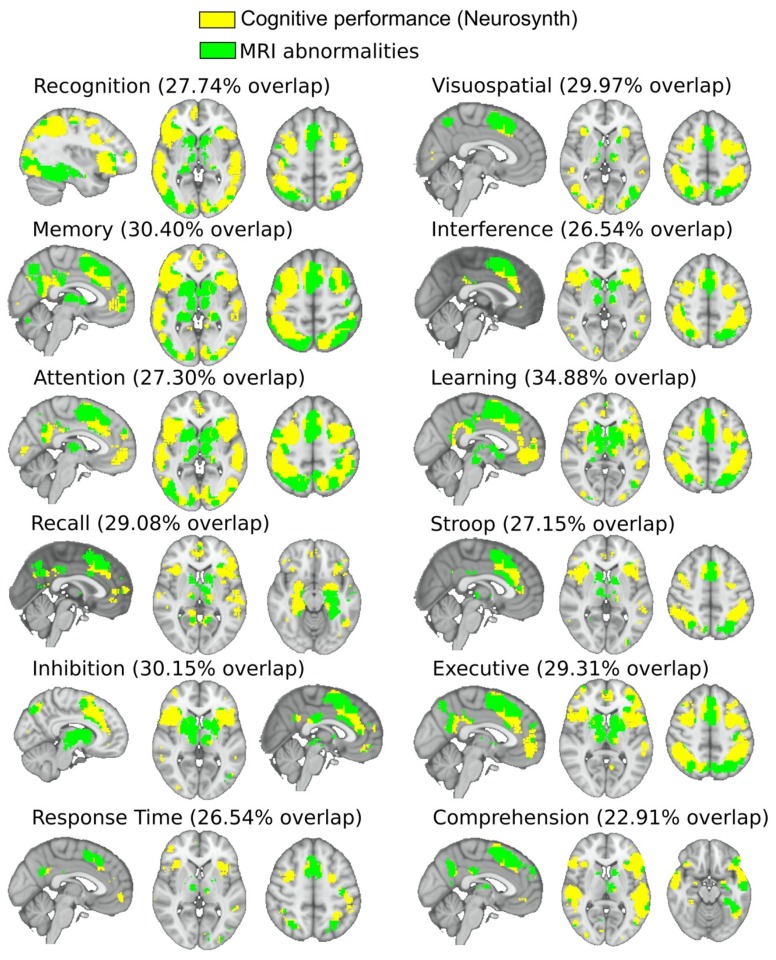
Correspondence between brain MRI alterations and cognitive performance in combined Cobalamin D disorder. By pooling together the brain maps proceeding from the three classes of imaging alterations represented in Figure 3 (colored in green), we illustrate their overlapping with cognitive maps (yellow color) obtained from Neurosynth co-activation maps.

This study assessed fMRI at rest. When looking to the intersection between neurosynth cognitive networks and brain MRI abnormalities resulting from either structural, or diffusion or functional images (Figure 4), we found that MRI abnormalities matched to co-activation maps resulting from task-fMRI studies in several paradigms such as recognition (overlapping index of 27.74%), visuospatial (29.97%), memory (30.40%), interference (26.54%), attention (27.30%), learning (34.88%), recall (29.08%), Stroop (27.15%), inhibition (30.15%), executive (29.31%), response time (26.54%), and comprehension (22.91%).

### 3.4. Neuropsychological Characterization

**Figure 5:**
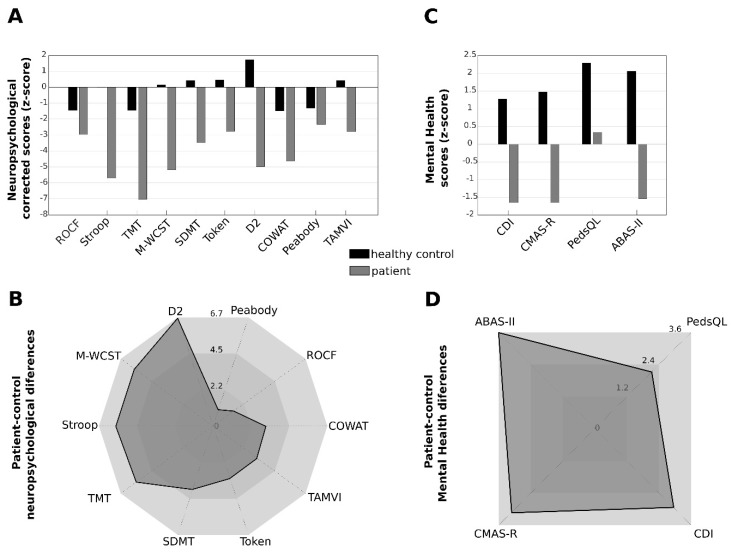
Neuropsychological and psychosocial alterations in combined Cobalamin D disorder: (**A**) corrected Z-scores for both patient (grey) and control (black) of ten different neuropsychological tests measuring different neuropsychological functions: Rey–Osterrieth Complex Figure Test (ROCF; visuoconstructive skill; memory), The Stroop Color and Word Test (Stroop; attention and inhibitory control), Trail Making Test (TMT; attention and mental flexibility), Modified Wisconsin Card Sorting Test (M-WCST; executive function), Symbol Digit Modalities Test (SDMT; processing speed), Shortened Version of the Token Test (Token; receptive language), D2 Test of Attention (D2; attention), Controlled Oral Word Association Test (COWAT; language and executive function), Peabody Picture Vocabulary Test (Peabody; vocabulary), Learning and Verbal Memory Test (TAMV-I; verbal memory); (**B**) patient-control differences for each of the ten different neuropsychological scores; (**C**) and (**D**) evaluation results and patient-control differences for tests assessing mental health, quality of life, and adaptive behavior: Children Depression Inventory (CDI; depression), Revised Children’s Manifest Anxiety Scale (CMAS-R; anxiety), Pediatric Quality of Life Inventory (PedsQL; quality of life), and Parent-Report, and Adaptive Behavior Assessment System-II (ABAS-II; adaptive behavior).

The results of the neuropsychological evaluation of the cblD patient and the healthy control, represented in age- and sex-standardized Z-scores, are shown in Figure 5. Overall, the MMA/HC cblD patient scored lower on all neuropsychological tests as compared with his twin sister. The patient’s Z-score values were <−2 in all of the tests and <−3 on six of them (Figure 5A). Major differences, represented in Figure 5B, were found on tests of attention (D2 test; with Z-score difference of 6.7), executive functioning (M-WCST; Z-score difference of 5.7), inhibitory control (Stroop; difference of 5.7), and mental flexibility (TMT; difference of 5.6). Similar differences were found also in the areas of mental health, quality of life, and adaptive behavior (Figure 5C,D) with the patient scoring lower than his sister, especially in adaptive behavior (ABAS-II; difference of 3.6), anxiety (CMAS-R; difference of 3.1), depression (CDI; difference of 2.9), and quality of life (PedsQL; difference of 2.0).

## 4. Discussion

The present study describes the clinical, genetic, neuroanatomical, neuropsychological, and psychosocial characteristics of a patient with late-onset MMA/HC cblD disorder as compared with healthy control, revealing severe morphological damage, brain circuit alterations, with clinically correlating neuropsychological impairments. To the best of our knowledge, this is the first time that brain functionality and brain circuit alterations are assessed in a patient with an inborn error of cobalamin metabolism, showing for the first time functional alterations in central nervous system motor tracts, thalamus, cerebellum, and basal ganglia in a patient with B12-related disorders.

Up to date, most patients described with the cblD disease presented a more severe phenotype with an early onset on the first days of life. This in contrast to milder forms of the disease, such as that of the patient described in this study, which is extremely rare. Until this study, specific circuit alterations underlying late-onset brain injury in patients with normal neurodevelopment have remained unknown. Brain circuits with major damage were found in the corpus callosum (affecting interhemispheric coherence), cerebellum (affecting majorly to movement control), and fronto-striatal-thalamic circuit (affecting the dopaminergic and cognitive control). At the morphological level, we showed an increase in ventricular volume of 254%, a value much higher than the one typically reported in other neurological disorders. In addition, we found cortical and subcortical atrophies, mainly at the precuneus and striatum.

The patient described in this manuscript presented a mild form of cblD disease with a clinical onset at 10 years of age. However, the severe alterations found in corpus callosum are typically associated with neurodevelopmental problems [54,55]. We suggest that early versus late onset may follow a transition, although the precise moment at which the disease starts to produce cognitive and neurological symptoms is not known. Similar to what has been suggested in other neurological disorders [56], late-onset neurological symptoms might initiate when the damage affects high-connectivity regions in the human connectome (also known as hubs), so after being damaged, their effects can easily propagate to the whole network. Such a possibility is consistent with the lesioned areas in our patient, which are well-identified hubs in the human brain [57], as it occurs, for instance, in subcortical hubs (such as the putamen and caudate) and cortical ones (such as the precuneus and anterior cingulate cortex).

The present study showed alterations in the corpus callosum, and other commissural tracts such as forceps minor and major, similar to previous findings in vitamin B12-deficient patients [58,59]. Importantly, we have shown that combined cblD defect produces alterations in motor tracts such as the corticospinal tract and cerebellar peduncle. Very few studies have found lesions in motor tracts in vitamin B12-deficient patients [60], and none of them found them in the commissural tracts, perhaps indicating a more severe brain damage in this inborn error of cbl metabolism as compared with nutritional/acquired vitamin B12 deficiency.

Perfusion studies (not assessed here) have shown that vitamin B12 deficiency produces decreased blood flow in occipital and parietal regions, and increased flow in frontal and temporal regions [61]. Although related, the perfusion signal is different from the BOLD signal studied here, the former related to arterial blood delivery and the latter to the total amount of blood deoxyhemoglobin. We have found alterations (as measured by variance increase in the BOLD signal) in both cortical (occipital and temporal) and subcortical regions (hippocampus, thalamus, cerebellum, and basal ganglia), in agreement with fMRI studies at rest in vitamin B12 deficiency showing alterations in temporal, parietal, and frontal cortices, together with the hippocampus [62]. However, as far as we know, no other studies in vitamin B12 deficiency have shown functional alterations in thalamus, cerebellum, and basal ganglia, suggesting that this inborn error of cbl metabolism, as compared with vitamin B12 deficiency, produces a different, more severe form of brain damage.

Although previous case reports of cblD defect patients have reported the presence of developmental delay, learning disabilities, and cognitive impairment [2,4,7,8,51,63,64], our study is the first one that includes a comprehensive neuropsychological evaluation to measure a variety of cognitive domains in a patient with the cblD disease. Our results show that the patient presented low scores in attention, memory, speed of information processing, inhibitory control, mental flexibility, and language measures, in accordance with studies with vitamin B12-deficient patients [58,65]. Moreover, the patient with MMA/HC cblD disorder showed poor psychosocial functioning in the areas of communication, academic skills, home life, leisure activities, and social life, as well as symptoms of anxiety and depression and low quality of life.

These results are consistent with those found in previous studies on cblD disease, in which behavioral problems such as aggression and hyperactivity [7,8], poor social skills [8], and psychiatric disorders and personality changes [2] have been found. Moreover, our study incorporates the characterization of the emotional and psychosocial functioning in cblD disease through quantified psychological scales, in contrast to previous studies where these symptoms were mentioned in a qualitative manner during the clinical description of the cases.

Low vitamin B12 and high homocysteine levels are common findings in the elderly [66], and some recent longitudinal studies have linked these analytical disturbances with an increased rate of brain atrophy [67,68] and progression of white matter lesion volume [69], accelerating aging and worsening cognitive decline. In addition, recent findings also suggest that the major circuit affected in physiological aging is the fronto-striato-thalamic circuit [20], in accordance with the results of this study. Moreover, our results on neuropsychological impairment are in accordance with task functional studies showing that attention, executive function, and mental flexibility are severely impaired during aging [70,71]. Although causality cannot be established in the studies assessing the relationship between vitamin B12 levels and aging, our study shows in a 12-year-old boy with an inborn error of cbl metabolism with low vitamin B12/high homocysteine levels, brain atrophy, brain circuits´ alterations, and neuropsychological disturbances similar to those described in old populations (and thus with multiple comorbidities working as possible confounders), re-enforcing the suggested role of vitamin B12 deficiency/dysfunction on neurological aging.

The main limitation of the present study is the sample size, which is due to the ultra-rare nature of the disease, where only five patients with late-onset MMA-HC cblD defects have been described so far. This limitation is compensated by the participation of the patient´s twin sister, a well-matched control as they both have been exposed to the same cognitive, social, and educational stimuli, and both exactly have the same age and years of education, all these variables being important cofounders in neuroimaging studies. Because of the sample size limitation, either group comparison or the association between brain circuit and cognitive function could not be assessed as is usually done in neuroimaging studies, where the group differences and association with test scores, or biochemical parameters, are assessed by fitting a general linear model to each image voxel or region of interest, followed by a multiple comparison correction. Instead, brain alterations in our study have been defined as the ratio of variations between the images belonging to the patient and control in the different modalities, and overlapped to brain maps obtained from different task functional studies, showing that in a qualitative manner brain imaging alterations matched to a plethora of different co-activation maps existing during cognitive tasks. In the future, we will assess the longitudinal variations of the different metrics analyzed here, all of them calculated as ratios of patient vs. control, determining whether or not there exists a recovery in the different studied domains, such as cognitive, emotional, and psychosocial functioning, but also in relation to structural imaging and brain circuit’s alterations.

## 5. Conclusions

This study assessed brain functionality and cognitive performance in inborn errors of cbl metabolism as late-onset MMA/HC cblD disorder, describing morphological damage (ventricular enlargement and atrophy), brain dysfunctionality, and circuit alterations affecting central nervous system motor tracts, thalamus, cerebellum, and striatum, linked to neuropsychological and psychosocial alterations, which will facilitate the expansion of the understanding of cognitive impairment in vitamin B12-related disorders.

## Figures and Tables

**Table 1:** Biochemical parameters at the diagnosis time before the treatment initiation (basal) and at the study visit.

Variables	Basal	Study Visit
Age (years)	11.4	11.8
Plasma homocysteine (µmol/L) (normal range: 5–15)	144	30.6
Serum methylmalonic acid (µmol/L) (normal range: 0.08 to 0.58)	67.8	5.3
Plasma methionine (µmol/L) (normal range: 7 to 47)	18	36
Plasma propionylcarnitine (µmol/L) (normal range: 0.05 to 0.5)	6.1	1.7
Serum cobalamin (pg/mL) (normal range: 220–980)	312	>2000

**Table 2:** *MMADHC* mutations and genotype in patients with MMA/HC cblD defect.

Patient Phenotype	Patient Genotype ^1^	Onset	Alelle 1	Protein Change Alelle 1 ^3^	Gene Location	Alelle 2	Protein Change Alelle 2	Gene Location	Ref.
MMA/HC	g1 (1)	Early	c.419dupA	Y140Xfs	Exon 5	Y140Xfs	Y140Xfs	Exon 5	[4]
MMA/HC	g5 (1)	Early	c.472C > T	R158X	Exon 5	c.472C > T	R158X	Exon 5	[50]
MMA/HC	g4 (1)	Early	c.683C > G	S228X	Exon 7	c.683C > G	S228X	Exon 7	[8]
MMA/HC	g2 (1)	Early	c.696 + 1_4delGTGA	F204_A232del (skip exon 7)	Intron 7	c.696 + 1_4delGTGA	F204_A232del (skip exon 7)	Intron 7	[4]
MMA/HC	g3 (5)	Late	c.748C > T	R250X	Exon 8	c.748C > T	R250X	Exon 8	[4,51,52,53]
MMA/HC	g6 (1) ^2^	Late	c.438_442delAGAGT	F161fsX14	Exon 5	c.748C > T	R250X	Exon 8	This study

^1^ Number of reported patients for each genotype (g1–g6) is indicated in brackets. ^2^ *MMADHC* variant in alelle 1 of the patient reported in this study has not been described before. Note that this patient is the only *MMADHC* heterozygous patient with MMA/HC cblD defect. ^3^ Amino acids are indicated by the single-letter code. Ref: references; del: deletion; dup: duplication; fs: frameshift; X: stop codon.
